# Further Studies on Antioxidant Potential and Protection of Pancreatic *β*-Cells by *Embelia ribes* in Experimental Diabetes

**DOI:** 10.1155/2007/15803

**Published:** 2007-04-12

**Authors:** Uma Bhandari, Neeti Jain, K. K. Pillai

**Affiliations:** Department of Pharmacology, Faculty of Pharmacy, Hamdard University, New Delhi 110062, India

## Abstract

This study was designed to examine the antioxidant defense by ethanolic extract of *Embelia ribes* on streptozotocin-(40 mg/kg, intravenously, single-injection) induced diabetes in Wistar rats. Forty days of oral feeding the extract (100 mg/kg and 200 mg/kg) to diabetic rats resulted in significant (*P* < .01) decrease in blood glucose, blood glycosylated haemoglobin, serum lactate dehydrogenase, creatine kinase, and increase in blood glutathione levels as compared to pathogenic diabetic rats. Further, the extract also significantly (*P* < .01) decreased the pancreatic thiobarbituric acid-reactive substances (TBARS) levels and significantly (*P* < .01) increased the superoxide dismutase, catalase, and glutathione levels as compared to above levels in pancreatic tissue of pathogenic diabetic rats. The islets were shrunken in diabetic rats in comparison to normal rats. In the drug-treated diabetic rats, there was expansion of islets. The results of test drug were comparable to gliclazide (25 mg/kg, daily), a standard antihyperglycemic agent. The study concludes that *Embelia ribes* enhances the antioxidant defense against reactive oxygen species produced under hyperglycemic condition and this protects *β*-cells against loss, and exhibit antidiabetic property.

## 1. INTRODUCTION

Diabetes mellitus, a global public health problem, is now emerging as a pandemic and by the year 2025, three quarters of the world's 300 million adults with diabetes will be in nonindustrialized countries, and almost a third in India and China alone [1].

Increased free radical generation and oxidative stress are hypothesized to play an important role in pathogenesis of diabetes and its late complications [2]. Possible sources of oxidative stress and damage to proteins in diabetes include free radicals generated by auto-oxidation reactions of sugars and sugars adducts to proteins and by auto-oxidation of unsaturated lipids in plasma and membrane proteins. The oxidative stress may be amplified by a continuing cycle of metabolic stress, tissue damage, and cell death, leading to increased free radical production and compromised free radical inhibitory and scavenger systems, which further exacerbate the oxidative stress [3]. Indeed, there is widespread acceptance of possible role of reactive oxygen species (ROS) generated as a result of hyperglycaemia in causing many of the secondary complications of diabetes such as nephropathy, retinopathy, neuropathy [4], and cardiomyopathy [5]. Glycation reaction in diabetes occurs in various tissues including *β*-cells [6, 7]. The activity of antioxidant enzymes such as superoxide dismutase, catalase, and glutathione peroxidase, which is low in islet cells when compared to other tissues, becomes further worsened under diabetic conditions [8]. Further, the presence of higher glucose or glycated protein concentration enhances lipid peroxidation [9], and furthermore lipid peroxides may increase the extent of advanced glycation end products [10].

The pharmacotherapy of diabetes has recently undergone unprecedented expansion. However, the challenge is to optimize glycaemic control with minimum number of medication while taking into consideration the cost of the therapy, adverse effect profiles, ease of administration, and the urgency for blood sugar normalization.

Recent awareness of therapeutic potential of several traditionally used plants has opened a new dimension for the study and research of medicinal plants. In traditional medicine, several Indian medicinal plants or their extracts have been used to treat diabetes [11].

*Embelia ribes burm* (family, *Myrsinaceae*) known commonly as *vidanga* is widely distributed throughout India. It is highly esteemed in Ayurveda as a powerful anthelmintic [12] and also an important ingredient of a number of formulations [13, 14]. Ayurveda also describes *vidanga* as pungent and cures flatulence and colic. In a preliminary study, Tripathi [15] reported the antihyperglycemic activity of decoction of the *E. ribes* fruits in glucose-fed albino rabbits. Further, Bhandari et al. [16] reported the potential of ethanolic extract of *Embelia ribes* in diabetic dyslipidemia and protection from lipid peroxidation in tissues in streptozotocin-induced diabetes in rats (20 days' study).

The present study is a further attempt in this direction. In the present study, the effect of 40 days' chronic oral treatment with ethanolic extract of *E. ribes* (100 mg/kg and 200 mg/kg) on the basal level of some key serum and tissue antioxidants was investigated in streptozotocin-induced oxidative damage in rats.

## 2. MATERIAL AND METHODS

### 2.1. Preparation of the extract

Dried *E. ribes* fruits, 200 g, were purchased locally from a grocery shop in New Delhi (in India, it is commonly available) and authenticated by Dr. M. P. Sharma, Taxonomist, Department of Botany of our Institute. A voucher specimen was retained in the department (UB# 04).

The fruits were Soxhlet extracted with 90% ethanol for 72 hours. The solvent was removed under reduced pressure to give a dried extract, 7.5% yield w/w (with respect to the crude material), and two doses equivalent to 100 mg and 200 mg of the crude drug per_kilogram body weight were calculated, and suspended in 1%v/v tween 80 solution for the experiment.

### 2.2. Experimental induction of diabetes

The study was approved by Institutional Animal Ethics Committee (IAEC) (Registration no. and Date of Registration: 173/CPCSEA (Committee for the Purpose of Control and Supervision of Experiments on Animals), Government of India, dated 28th January, 2000).

Wistar rats of either sex (150 to 200 g) were obtained from the central animal house facility of Hamdard University, New Delhi. They were maintained under standard laboratory conditions at 25 ± 2°C, relative humidity (50 ± 15%) and normal photoperiod (12-hour light-dark cycle) were used for the experiment. Commercial pellet diet (MFD, by Nav Maharastra Chakan Oil Mills Ltd., New Delhi, India) and water were provided ad libitum.

After fasting 18 hours, the rats were injected intravenously through tail vein with a single dose of 40 mg/kg STZ (Sigma, St. Louis, Mo, USA), freshly dissolved in citrate buffer (pH 4.5). After injection, the rats had free access to food and water and were given 5% glucose solution to drink overnight to counter hypoglycaemic shock. Diabetes in rats were observed by moderate polydipsia and marked polyuria.

After 3 days, the fasting blood glucose levels were determined by orthotoluidine method [17]. The rats showing fasting blood glucose more than 200 mg/dL were considered diabetic and were selected for the experimentation [18].

### 2.3. Experimental design

Normal and diabetic rats (*n* = 10 each) were randomly divided into 5 groups of 10 rats each as follows:

- group 1: control rats given 1 mL of vehicle (1% tween 80) alone for 40 days;
- group 2: pathogenic diabetic rats (STZ treated only);
- group 3: STZ + ethanolic *E. ribes* extract treated (100 mg/kg);
- group 4: STZ + ethanolic *E. ribes* extract treated (200 mg/kg);
- group 5: STZ + gliclazide treated (25 mg/kg), a reference drug.

The test drug and reference standard drugs were fed orally for 40 days. Groups 1 and 2 rats received 1% tween 80 solution orally once a day for 40 days.

The experiment was terminated at the end of 40 days and the animals were fasted overnight.

### 2.4. Blood collection and biochemical estimations in serum

On 41st day, fasting blood samples were collected from the tail vein of all the groups of rats. Whole blood was collected for estimation of blood glucose [17], glycosylated hemoglobin (HbA_1C_) [19], and glutathione [20] levels.

Serum was separated for estimation of specific serum marker enzymes, namely, lactate dehydrogenase (LDH) [21, 22] and creatine kinase (CK) [23]. STZ-induced oxidative stress in diabetes is also a predictor of cardiac damage. Since LDH and CK are specific cardiac marker enzymes, increased serum LDH and CK levels were considered as marker of oxidative stress-induced cardiac damage.

### 2.5. Biochemical estimation in pancreatic tissue

After blood collection, all the animals were sacrificed and pancreas was dissected out. Tissue was washed with ice-cold saline, weighed and minced; 10% homogenate was prepared in 0.15 M ice-cold KCl for TBARS (thiobarbituric acid-reactive substances), a marker for lipid peroxidation [24] and protein estimation [25]; in 0.02 M EDTA for glutathione estimation [26]; and in phosphate buffer (pH 7.4) for superoxide dismutase (SOD) [27] and catalase estimations [28] using a Teflon tissue homogenizer. Decrease in levels of endogenous antioxidants with rise in TBARS levels was considered as oxidative stress.

### 2.6. Histological section of the pancreas

Pancreatic tissue was fixed in 10% formalin, routinely processed and embedded in paraffin wax. Paraffin sections (5 *μ*m) were cut on glass slides and stained with hematoxylin and eosin (H and E) and were examined under a light microscope by a pathologist blinded to the groups studied.

### 2.7. Statistical analysis

Statistical analysis was carried out using GraphPad Prism 3.0 (GraphPad software: San Diego, Calif). All data were expressed as mean ± SEM. Groups of data were compared with an analysis of variance followed by Dunnett's *t* test. Values were considered statistically significant, when *P* < .01.

## 3. RESULTS

Table 1 shows the levels of blood glucose and glycated hemoglobin in normal and experimental rats. The levels of glucose and glycated hemoglobin were elevated significantly in the group 2 diabetic control rats. After treatment with ethanolic extract of *E. ribes*, the levels of glucose and glycated hemoglobin were significantly lowered in both doses. Further, Table 1 shows the levels of serum marker antioxidant enzymes (LDH, CK and glutathione). The levels of blood glutathione in diabetic rats (group II) were significantly lowered (*P* < .01) when compared with those in normal control rats of group 1. Treatment with ethanolic *E. ribes* (100 mg/kg and 200 mg/kg) for 40 days significantly restored the blood GSH levels as compared to group II rats. Gliclazide treatment did not show any significant increase in blood GSH levels when compared to group 2.

Furthermore, the levels of other marker enzymes, that is, LDH and CK were significantly increased in group 2 diabetic rats. However, the test drug treatment for 40 days significantly reduced the levels of LDH (*P* < .05 with 100 mg/kg, i.e., group 3; *P* < .01 with 200 mg/kg, i.e., group 4) when compared to pathogenic diabetic rats.

Similarly, the administration of ethanolic extract of *E. ribes* significantly reduced (*P* < .01) the serum CK levels in both doses when compared to pathogenic diabetic control rats and the results were comparable to gliclazide treatment (group 5).

Table 2 presents the activities of the antioxidant enzymes in the pancreatic tissues in the control as well test drug-treated animals. Significant reduction in the activity of SOD in pancreas of diabetic animals (group 2) was observed in comparison to normal rats, that is, group 1. Diabetic rats treated with ethanolic extract of *E. ribes *(100 mg/kg and 200 mg/kg) showed normal enzymatic activity.

Catalase activity was significantly (*P* < .01) decreased in diabetic animals as compared to normal control rats, that is, group 1. The levels were significantly (*P* < .01) increased with ethanolic extract of *E. ribes *(in a dose of 200 mg/kg).

Total glutathione activity was reduced by 69.13% in pancreatic tissue of diabetic rats as compared to normal control animals. The levels were significantly (*P* < .01) increased with ethanolic extract of *Embelia ribes* (in a dose of 200 mg/kg).

### 3.1. Histopathological examination

**Figure 1:**
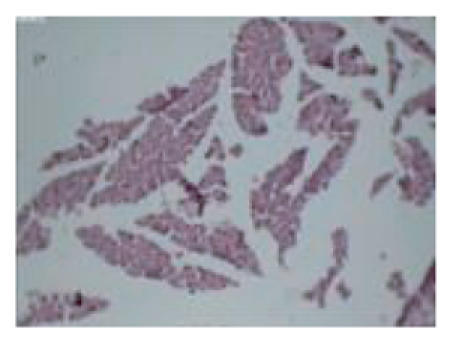
Typical photomicrograph of the pancreas of normal control rats (group 1), H and E × 10 shows normal islets.

**Figure 2:**
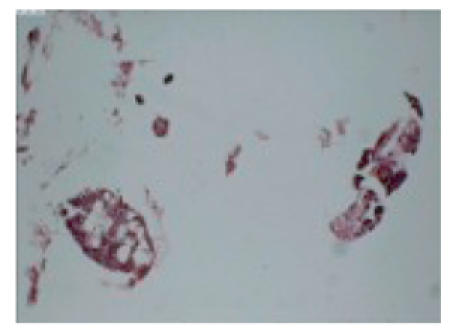
Typical photomicrograph of the pancreas of pathogenic diabetic (STZ only) control rats (group 2), H and E × 10 shows shrunken islets.

**Figure 3:**
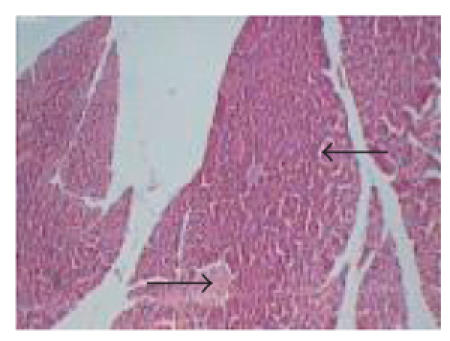
Typical photomicrograph of the pancreas of STZ + *Embelia ribes* (100 mg/kg) treated rats (group 3), H and E × 10 shows congested acinis.

**Figure 4:**
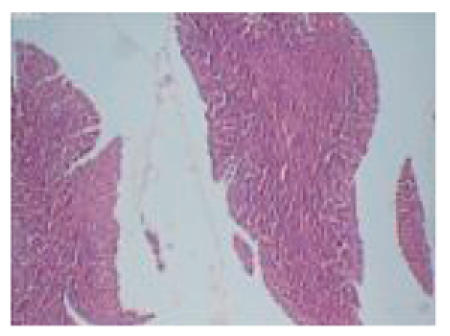
Typical photomicrograph of the pancreas of STZ + *Embelia ribes* (200 mg/kg) treated rats (group 4), H and E × 10 shows normal pancreatic islets.

**Figure 5:**
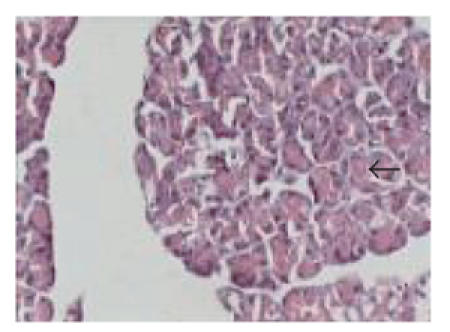
Typical photomicrograph of the pancreas of STZ + gliclazide-treated rats (group 5), H and E × 10 shows moderate expansion of islets.

Figures 1–5 depict the islet cells of the pancreas of rat in different groups. Figure 1 shows globules of acini with normal islet cells. The atrophy of islets cells with inflammatory infiltrate with edema was observed in group 2 diabetic rats when compared to group 1 control rats. Treatment of diabetic rats with the test drug in group 3 showed islet cells with congested acinis. However, group 4 treatment showed normal pancreatic cells, and gliclazide treatment in group 5 showed moderate expansion of islets cells.

## 4. DISCUSSION

The islet *β*-cells are susceptible to damage caused by oxygen-free radicals [29] since the antioxidant defense system is weak under diabetic condition [30]. The levels of antioxidant defense system are altered in STZ-induced diabetic rats, which is in good correlation with the present observation [31]. Nonprotein thiols like glutathione are one of the important primary defenses that counteract the oxidative stress. We observed lower levels of serum glutathione in STZ diabetic rats, which is in consistent with earlier reports [31, 32]. The observed decrease may be due to utilization of nonprotein thiols by increased oxygen-free radicals produced in hyperglycemic conditions associated with diabetes mellitus.

Increased serum CPK and LDH levels in diabetic rats indicate cardiac muscular damage [33]. Similar increase in the activity of these two enzymes in serum of the STZ diabetic rats was observed in the present study. The quantity of enzyme released from the damaged tissue is a measure of the number of necrotic cells [34].

Further, STZ-treatment in animals decreased the activity of marker enzymes in pancreatic tissue. SOD is an important defense enzyme which catalyzes the dismutation of superoxide radicals [35]. Catalase is a hemoprotein which catalyzes the reduction of hydrogen peroxides and protects the tissues from highly reactive hydroxyl radicals [36]. Therefore, reduction in the activity of these enzymes (SOD, CAT) results in a number of deleterious effects due to the accumulation of superoxide anion radicals and hydrogen peroxides. Lipid peroxidation is one of the characteristic features of chronic diabetes [37]. In the present study, a marked increase in the concentration of TBARS was observed in the pancreatic tissue of diabetic rats. Higher levels of lipid peroxides and low SOD and CAT activity indicate an oxidative stress condition.

The ethanolic extract of *Embelia ribes *produced a marked decrease in blood glucose levels at 100 mg/kg and 200 mg/kg body weight in STZ-diabetic rats after 40 days treatment. The antidiabetic effect of *Embelia ribes *may be due to increased release of insulin from the existing *β*-cells of pancreas similar to that observed after gliclazide administration.

Treatment of diabetic rats with the ethanolic extract of *Embelia ribes *significantly increased the levels of nonprotein thiols in serum as well as in pancreatic tissues of rats as compared to pathogenic diabetic rats. It has been reported by Sreepriya and Bali [38] while studying liver cancer protecting effect of Embelin, a constituent of *Embelia ribes* in rats, that Embelin significantly scavenges free radicals; and resulting in hepatic glutathione antioxidant defense decreases lipid peroxidation and minimizes the histological (liver) alterations induced by N-nitrosodiethylamine (200 mg/kg, single intraperitoneal (IP) injection) and phenobarbital (0.05% in drinking water) fed orally for 13 weeks [38]. In our study, we also observed significant (*P* < .01) increase in levels of blood glutathione, as well as pancreatic levels of glutathione in diabetic rats when treated with ethanolic extracts of *Embelia ribes*. Further, the activities of SOD and CAT were also increased in the pancreatic tissues of test drug-treated diabetic animals. The antioxidant activity of the test drug might have been due to the inhibition of glycation of the antioxidant enzymes SOD and CAT. Glucose which forms Schiff's base with proteins has been reported to have high affinity for proteins especially those containing transition metal ions [39]. Increased glycated Cu-Zn-SOD has been reported in diabetes. A study on curcumin (active principle of rhizome of *Curcuma longa*) has shown that curcumin inhibits advanced glycation end products in STZ diabetes in rats [40]. In the present study too, we observed a decrease in the glycated hemoglobin in rats treated with the ethanolic extract of *Embelia ribes *which is not reported earlier. Since the level of glycosylated hemoglobin has been shown to provide an index of blood glucose concentration during the previous 1-2-month period, it is being used increasingly in the clinical management of diabetes [41].

Furthermore, there was a significant attenuation of serum LDH and creatine kinase levels with the test drug treatment indicating the cardioprotective effect of ethanolic extract of *Embelia ribes*.

In the *Embelia ribes* 200 mg/kg dose, significant protection against STZ-induced oxidative stress was observed. The treatment showed normal pancreatic *β*-cells (Figure 4). The protection might have been mediated through an *Embelia ribes*-induced increase in basal pancreatic SOD and catalase activities.

Hence, we conclude that the ethanolic extract of *Embelia ribes* offers protection of *β*-cells against reactive oxygen species-mediated damage by enhancing cellular antioxidant defense and reducing hyperglycaemia in chemically induced diabetes.

## Figures and Tables

**Table 1:** Effect of ethanolic extract of *Embelia ribes* (ER) on whole blood glucose, whole blood glycosylated hemoglobin (HbA_1c_), blood glutathione (GSH), serum creatine kinase (CK), serum lactate dehydrogenase (LDH) in albino rats (*n* = 8).

Groups	Parameters				

	Blood glucose (mg/dL)	Whole blood HbA_1c_(%)	Blood GSH (mg/dL)	Serum CK (IU/L)	Serum LDH (IU/L)

I (Normal healthy control)	69.3 ± 0.78	5.055 ± 0.249	3.205 ± 0.074	60.74 ± 3.1	191.11 ± 7.40
II (STZ treated, i.e., pathogenic control)	344 ± 11.34*	18.50 ± 0.611*	1.115 ± 0.077*	235.35 ± 4.81*	477.17 ± 34.86*
III (STZ ± ER-100 mg/kg)	104.7 ± 1.84^#^	13.58 ± 0.239^#^	2.69 ± 0.2205^#^	183.13 ± 7.9^#^	375.5 ± 23.92^@^
IV (STZ ± ER-200 mg/kg)	87.7 ± 1.84^#^	11.62 ± 0.554^#^	4.147 ± 0.225^#^	71.85 ± 84^#^	273.84 ± 26.47^#^
V (STZ ± gliclazide-25 mg/kg)	79.05 ± 1.261^#^	8.81 ± 0.647^#^	2.082 ± 0.336	79.84 ± 4.42^#^	315.0 ± 0.31^#^

*P < .01, as compared to group I (ANOVA followed by Dunnett's *t* test).

^#^ *P* < .01, as compared to group II (ANOVA followed by Dunnett's *t* test).

^@^ *P* < .05, as compared to group I (ANOVA followed by Dunnett's *t* test).

**Table 2:** Effect of ethanolic extract of *Embelia ribes* (ER) on lipid peroxides (TBARS), catalase (CAT), superoxide dismutase (SOD), and glutathione (GSH) levels in pancreatic tissue of albino rats (*n* = 8).

Groups	Parameters			

	TBARS (nmol MDA/mg protein)	SOD (IU/mg protein)	CAT (nmol H_2_O_2_-consumed/min/mg protein)	GSH (*μ*mol of phosphorus liberated/min/mg protein)

I (Normal healthy control)	0.469 + 0.046	3.92 + 0.075	4.80 + 0.089	56.26 + 2.860
II (STZ treated, i.e., pathogenic control)	6.802 + 0.895*	0.162 + 0.030*	0.323 + 0.010*	17.35 + 5.020
III (STZ ± ER-100 mg/kg)	3.78 + 0.833^#^	3.937 + 0.263^#^	2.767 + 0.770	34.20 + 3.500^@^
IV (STZ ± ER-200 mg/kg)	0.488 + 0.065^#^	2.236 + 0.066^#^	4.67 + 0.901^#^	39.20 + 1.070^#^
V (STZ ± gliclazide-25 mg/kg)	2.159 + 0.401^#^	3.62 + 0.475^#^	4.66 + 1.320^#^	46.1 + 2.400^#^

*P < .01, as compared to group I (ANOVA followed by Dunnett's *t* test).

^#^ *P* < .01, as compared to group II (ANOVA followed by Dunnett's *t* test).

^@^ *P* < .05, as compared to group I (ANOVA followed by Dunnett's *t* test).
